# Unique antibodies across the animal kingdom (birds, camelids, and sharks): therapeutic potential against human respiratory viral infections

**DOI:** 10.3389/fimmu.2025.1723343

**Published:** 2025-11-21

**Authors:** Ammar A. Basabrain, Thamir A. Alandijany

**Affiliations:** 1 Department of Medical Laboratory Sciences, Faculty of Applied Medical Sciences, King Abdulaziz University, Jeddah, Saudi Arabia; 2 Hematology Research Unit, King Fahd Medical Research Center, King Abdulaziz University, Jeddah, Saudi Arabia; 3 Special Infectious Agents Unit, King Fahd Medical Research Center, King Abdulaziz University, Jeddah, Saudi Arabia

**Keywords:** IgY, nanobody, VHH, vNAR, IgNAR, respiratory viral infections, immunotherapy, influenza

## Abstract

Antibodies represent indispensable tools in the armamentarium against infectious diseases, with widespread application in prophylactic, therapeutic, and diagnostic settings. Conventional mammalian immunoglobulin G (IgG) antibodies have been extensively utilized in clinical and research contexts; however, their utility is sometimes constrained by intrinsic limitations such as thermal instability, susceptibility to proteolytic degradation, limited mucosal efficacy, and the high costs associated with mammalian expression systems. These challenges have driven increasing interest in alternative antibody formats derived from non-mammalian species that offer distinct structural and functional advantages. In recent years, a growing body of research has focused on non-canonical immunoglobulins, including immunoglobulin Y (IgY) from birds, nanobodies derived from the variable domain of heavy-chain-only antibodies (VHH) in camelids, and variable new antigen receptors (VNARs) sourced from the immunoglobulin new antigen receptor (IgNAR) system in cartilaginous fish such as sharks. The structural simplicity and functional robustness of these antibody platforms enable their integration into diverse biomedical applications, encompassing passive immunization, targeted drug delivery, and point-of-care diagnostics. Indeed, these molecules exhibit unique biochemical properties, including superior thermal and protease resistance, small molecular size, and the ability to access recessed or conformational epitopes that are often inaccessible to conventional IgG antibodies. Moreover, their typically lower immunogenic profiles and reduced pro-inflammatory activity render them suitable for a broad range of therapeutic strategies, including repeated administration and mucosal delivery, and position them as particularly promising agents for combating respiratory pathogens. This review highlights the unique properties, practical advantages, and translational therapeutic potential of IgY, nanobodies, and VNARs. It underscores their advantages over traditional antibody formats and their emerging role as next-generation Immunotherapeutics in the global effort to address persistent and emerging respiratory viral threats.

## 1 Introduction

Antibodies have long stood as one of the most powerful tools in immunotherapy, offering targeted mechanisms to neutralize pathogens, modulate immune responses, and enable precision diagnostics (1). Among them, monoclonal antibodies (mAbs) derived from mammalian immunoglobulin G (IgG) have revolutionized the treatment of infectious diseases, autoimmune conditions, and cancers (2–4). Their ability to selectively bind and inactivate viral and microbial antigens has led to several approved therapeutics and widespread clinical success. However, despite their achievements, IgG-based therapies are not without limitations. High production costs, complex manufacturing, limited stability in mucosal environments, and Fc-mediated inflammatory responses pose considerable challenges, especially for large-scale or field-deployable applications (2–4).

**Figure 1:**
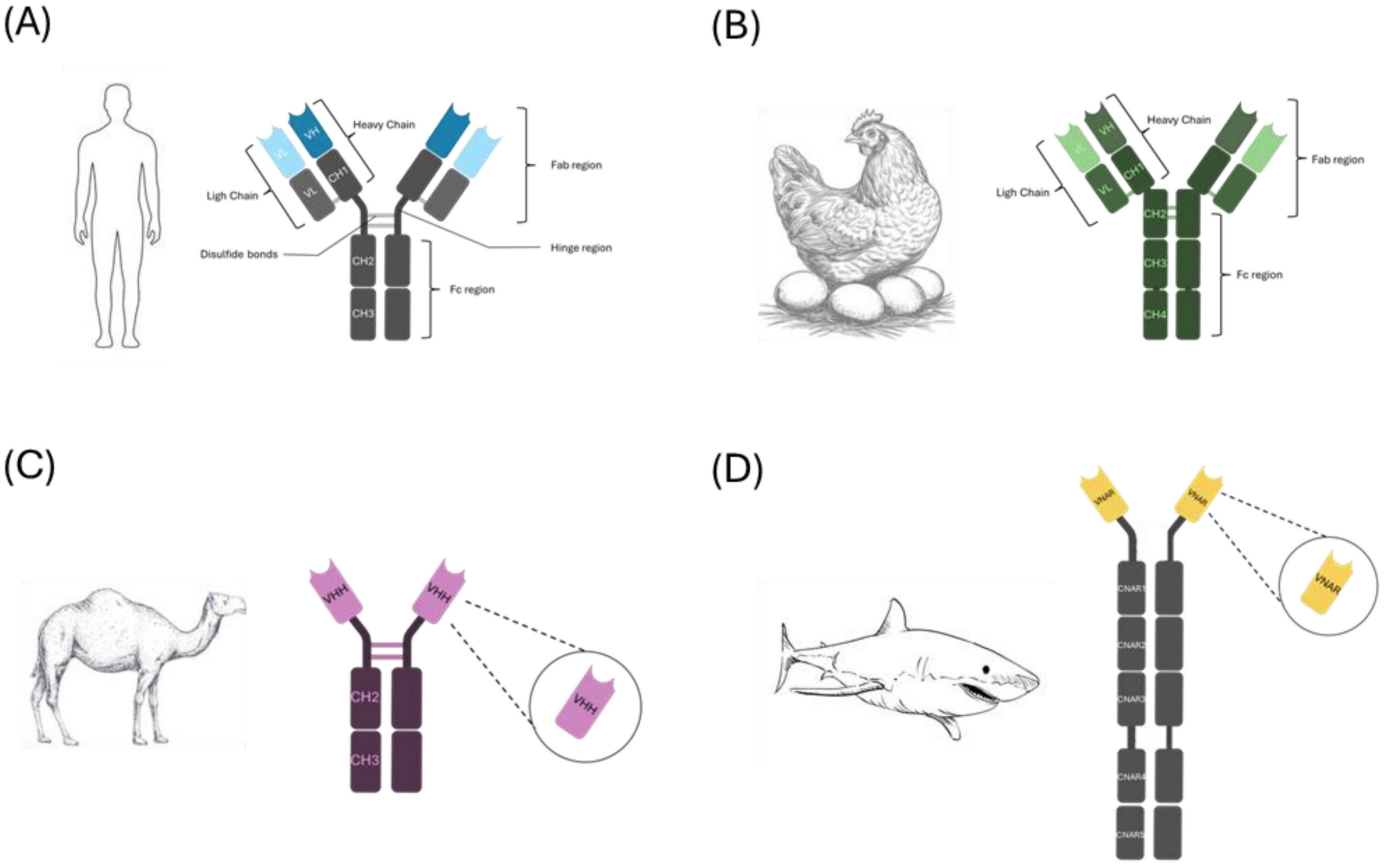
Structural diversity of antibodies across different species. This diagram illustrates the immunoglobulin (Ig) structures from various vertebrates, highlighting key differences in their architecture. **(A)** Human IgG (top left): Composed of two heavy chains and two light chains, forming a Y-shaped molecule with CH1, CH2, and CH3 constant domains, and variable VH and VL regions responsible for antigen binding. **(B)** Chicken IgY (top right): Structurally similar to IgG but with a longer Fc region that includes CH1–CH4 domains, lacking the hinge region seen in mammalian IgG. **(C)** Camelid heavy-chain antibody (bottom left): Consists solely of heavy chains without light chains, containing CH2 and CH3 domains and a single variable domain (VHH or nanobody) that mediates antigen binding. **(D)** Shark IgNAR (bottom right): Composed of heavy chains only, with a unique structure characterized by multiple constant domains (CNAR1–CNAR5) and a single variable domain (VNAR) at the N-terminus.

In response to these limitations, growing attention is being directed toward unconventional antibodies produced by non-mammalian species. Animals such as birds, camelids, and sharks have evolved unique immunoglobulin types, IgY, heavy-chain-only antibodies (HCAbs), and IgNAR, respectively, with distinctive structural and functional traits (5–8). These extraordinary antibodies offer several advantages over classical IgG, including enhanced biochemical stability, reduced immunogenicity, superior tissue penetration, and the ability to bind novel or cryptic epitopes inaccessible to conventional antibodies (5–8) (**Figure 1**). For instance, IgY antibodies from chicken egg yolk can be harvested non-invasively and have shown promise in mucosal protection and passive immunization (5, 9). Nanobodies, derived from the variable domain of camelid HCAbs, are small, single-domain fragments that can be engineered for high affinity and deep tissue penetration (6, 8, 10). Shark variable new antigen receptors (VNAR) derived from IgNAR antibodies represent an ancient yet highly stable form of immunity that provide evolutionary insights for therapeutic design (7, 11).

This review explores the therapeutic potential of these unconventional antibodies in combating respiratory viral infections. By highlighting both their strengths and translational potentials, this review aims to shed light on how these lesser-known unique immunoglobulins may help shape the next generation of antibody-based therapeutics.

## 2 IgY (avian-derived antibodies in egg yolk)

While most antibody therapies in modern medicine are based on mammalian IgG, alternative immunoglobulins found in birds have steadily gained attention, especially due to their accessibility and distinct immunological properties (1, 3). Birds have developed unique mechanisms for generating antibody diversity, primarily relying on gene conversion, rather than somatic hypermutation as seen in mammals (12).

Chief among these is Immunoglobulin Y (IgY), the major serum antibody in avian species. From an evolutionary standpoint, IgY is considered an ancient precursor to IgG (13). However, unlike IgG, which is predominantly found in mammalian plasma, IgY is naturally concentrated in egg yolk, a reflection of the bird’s strategy to confer passive immunity to its offspring during early development (14). IgY antibodies derived from egg yolk offer a range of unique properties and practical advantages that make them a compelling alternative to mammalian IgG for passive immunization strategies.

### 2.1 Unique properties of IgY: comparative advantages of avian IgY over mammalian IgG

Structurally, IgY differs in notable ways from its mammalian analogues (15). It is heavier in molecular weight, has a different glycosylation pattern, and lacks the hinge region found in IgG as supported by biophysical analyses (15, 16) (**Figure 1**). While this reduces conformational flexibility, IgY displays greater stability under harsh conditions and enhance its resilience during storage and formulation, but with less flexibility in antigen binding (17). More importantly, IgY does not bind to mammalian Fc receptors and does not activate the complement system, key factors that help minimize inflammatory responses and enhances its safety for therapeutic use (18, 19). Indeed, IgY’s lack of interaction with mammalian Fc receptors and complement proteins making it an excellent candidate for passive immunization, particularly in immune-compromised individuals or in mucosal applications where local inflammation could be detrimental (9, 18–20). In addition, this makes IgY ideal for diagnostic applications, especially in settings where interference from host IgG or rheumatoid factor can confound results (21, 22).

Immunologically, IgY benefits from its phylogenetic distance from mammals (15). Hens produce strong humoral responses after just two immunizations, generating robust quantities of specific antibodies that remain present in the yolk for several months (23, 24). This heightened responsiveness often results in greater antigen recognition breadth, especially for mammalian proteins that may be poorly immunogenic in mammals themselves (25).

Functionally, IgY has a superior stability profile (26). It demonstrates enhanced resistance to proteolytic enzymes, retaining significant biological activity even after prolonged exposure to gastrointestinal proteases such as trypsin and chymotrypsin (27). This property is especially important for oral delivery routes, where mammalian IgG would typically degrade before reaching the target site.

### 2.2 Practical advantages of IgY production

What makes IgY particularly appealing is not just its biological role but also its practical advantages (5). It can be harvested directly from eggs, offering a non-invasive and animal-friendly production system (28). This contrasts with traditional IgG extraction, which involves repeated blood draws from animals. From a production standpoint, IgY is remarkably cost-effective. A single hen can produce over 22 grams per year of total IgY annually, with 2% to 10% of that being antigen-specific (28). This yield is equivalent to the amount of IgG obtained from approximately four to five rabbits, but without the need for blood collection or animal sacrifice (28).

Although the current biopharmaceutical market is dominated by monoclonal IgG-based drugs, the technological and commercial potential of IgY is steadily expanding. Its production simplicity, ethical sourcing, and low-cost scalability, paired with its broad utility in diagnostics and promising results in infectious disease models, suggest that IgY is not just an academic novelty, but a serious candidate in the future landscape of immunotherapy.

### 2.3 Potential therapeutic applications of IgY against respiratory viruses

The physicochemical resilience of IgY, being stable across pH 3.5–11 and moderately resistant to pepsin degradation, makes it particularly well suited for formulations such as nasal drops, mouth rinses, and enteric-coated capsules designed to target mucosal surfaces where many respiratory viruses initially establish infection (20, 29). Recent formulation advances using alginate microspheres, chitosan nanoparticles, and lipid carriers can further extend IgY stability and bioavailability, particularly for aerosolized or lyophilized forms suitable for field or outpatient use. Hence, there is growing interest in utilizing IgY for therapeutic use, particularly for mucosal infections (**Table 1**). Since it shows good stability in gastrointestinal environments and does not elicit strong immune reactions when administered orally or nasally, it holds promise for passive immunization against respiratory viruses such as influenza viruses, coronaviruses (MERS-CoV and SARS-CoV-2), and Bovine respiratory syncytial viruses (BRSV).

**Table 1:** Summary of avian IgY antibodies developed against respiratory viruses, highlighting their prophylactic and therapeutic applications.

Study/year	Viral target	Igy target antigen	Treatment timing	Outcome/notes
Nguyen et al., 2010 (29)	Influenza A (H5N1, H1N1)	Hemagglutinin (HA)	Prophylactic & therapeutic	Significant reduction in lung viral titers
Wen et al., 2012 (30)	Influenza B virus	Hemagglutinin (HA)	Prophylactic & therapeutic	Reduced viral replication in lungs
Yang et al., 2014 (31)	Influenza A (H1N1)	Hemagglutinin (HA)	Prophylactic & therapeutic	Reduced viral titers in lungs
Abbas et al., 2020 (32)	MERS-CoV	Spike S1	Prophylactic/Therapeutic (*in vitro* ± *in vivo* basis)	Decreased infectivity *in vitro*; basis for *in-vivo* protection
El-Kafrawy et al., 2021 (33)	MERS-CoV	Full-length Spike (S)	Therapeutic (post-infection; hDPP4-Tg mice)	IC_50_≈ 51 µg/mL; ↓ antigen-positive lung cells & inflammation
Bao et al., 2021 (34)	SARS-CoV-2 (S1)	Spike S1	Prophylactic (*in vitro*/pseudovirus)	Blocked S1–ACE2 binding; IC_50_≈ 0.99 mg/mL
Agurto-Arteaga et al., 2022 (35)	SARS-CoV-2	Receptor-Binding Domain (RBD)	Prophylactic & Therapeutic (intranasal)	Reduced lung pathology & improved clinical parameters
El-Kafrawy et al., 2022 (36)	SARS-CoV-2 (RBD)	RBD	Prophylactic (intranasal, mice)	↓ lung viral replication; ↓ inflammation/edema/hemorrhage
Frumkin et al., 2022 (37)	SARS-CoV-2 (RBD)	RBD	Prophylactic (intranasal; hamster pilot + Phase 1 safety)	Broad variant neutralization; safe in humans (no systemic absorption)
Madai et al., 2024 (38)	SARS-CoV-2	Spike S1/RBD	Prophylactic (intranasal, hamsters)	↓ lung viral load and pathology
Zhao et al., 2024 (39)	SARS-CoV-2 (RBD, incl. Omicron BA.2.76)	RBD	Prophylactic/Therapeutic (hamsters)	~1-log₁₀ ↓ lung viral RNA; protection from weight loss & pathology

Preclinical studies have shown that chickens immunized with inactivated influenza viruses produce high titers of functional IgY in egg yolks (40). For instance, immunization with H1N1 resulted in IgY that bound strongly to viral hemagglutinin and neuraminidase, as confirmed by hemagglutination inhibition and Western blot assays (40). These IgYs reduced viral infection in plaque reduction assays and protected mice from lung pathology and weight loss when administered intranasally (40). In another study targeting influenza B, virus-specific IgY was produced in large quantities (over 75 mg per yolk) with high purity (30). *In vitro* assays showed the IgY neutralized infection in MDCK (Madin-Darby Canine Kidney) cells, while *in vivo* intranasal administration in mice reduced viral replication and provided effective protection (30). These findings support the idea that intranasal IgY delivery could be a viable tool in managing influenza outbreaks, particularly in situations where vaccine production lags behind viral evolution.

IgY antibodies have also demonstrated cross-reactivity and protection against multiple influenza strains. Mice treated intranasally with IgY against H5N1 showed full protection against not only H5N1 but also the A/Puerto Rico/8/34 (H1N1) strain. Additionally, prophylactic and post-infection treatment with these antibodies conferred complete recovery in cases of lethal H5N1 and H5N2 challenges (41). Notably, trace levels of anti-H5N1 IgY have even been detected in commercially available eggs, highlighting the robustness of the immune response in hens (29). Expanding beyond chickens, ostriches have been used as an IgY source, with immunization against swine influenza virus strains yielding substantial amounts of cross-reactive IgY (40). These antibodies demonstrated strong activity against both pandemic and swine-origin H1N1 strains, as shown by ELISA, immunocytochemistry, and viral neutralization assays (40). The high yield per bird and cost-effectiveness of ostrich IgY production make this an attractive option for large-scale antibody manufacturing during pandemics. Avian species diversity (e.g., ducks, turkeys, and quails) can be a biotechnological advantage for their egg-derived immunoglobulins, some of which may show unique glycosylation patterns that enhance mucosal adhesion and viral-neutralization efficiency. Such interspecies comparisons could inform next-generation scalable antibody-farming systems.

During the 2002–2003 SARS outbreak, hens were immunized with recombinant SARS-CoV spike (S) protein antigens, generating IgY that neutralized the virus (42). These antibodies retained functionality after lyophilization, supporting their potential for long-term storage and emergency distribution (42). For SARS-CoV-2, hens were similarly immunized with the receptor-binding domain (RBD) of the S protein (36). The resulting IgY was shown to competitively inhibit the interaction between viral RBD and human ACE2 receptor *in vitro*. Mouse models challenged with SARS-CoV-2 confirmed that intranasal IgY administration conferred protection, reducing viral load and pathological features in the respiratory tract (36). A phase I clinical trial using nasal IgY spray reported no adverse events, though large-scale efficacy data are still awaited (43, 44). Other groups have expanded this evidence base: intranasal or oral IgY formulations targeting the SARS-CoV-2 RBD demonstrated cross-neutralization against Alpha, Beta, Delta, and Omicron variants, significantly lowering lung viral RNA copies and preventing alveolar damage in animal models (36, 37, 39). The absence of systemic absorption following intranasal use underscores its safety, and the ability to rapidly adapt IgY production to new spike variants suggests an agile, variant-agnostic passive immunotherapy approach (35, 38). Furthermore, MERS-CoV studies confirmed that IgY raised against the S1 or full-length spike protein achieved potent neutralization (IC_50_≈ 51 µg/mL) and reduced histopathological lung inflammation in hDPP4-transgenic mice (32, 33). Together, these coronavirus-targeted IgY investigations establish a conceptual bridge between zoonotic coronaviruses and human passive-immunization platforms, positioning IgY as a first-line rapid countermeasure in future coronavirus spillovers.

Bovine respiratory syncytial virus (BRSV) shares a close genetic and pathogenic resemblance to human RSV, a major cause of lower respiratory infections in children, particularly under five years of age. There is a lack of broadly effective treatments for RSV. BRSV in calves serves as a suitable animal model for preclinical testing of novel therapeutics. In a comparative study, hens immunized with different BRSV doses produced IgY antibodies that showed high neutralizing titers *in vitro* (45). Notably, a simplified two-dose regimen yielded comparable results to more extended immunization schedules, suggesting that increasing antigen concentration can reduce the number of injections needed—benefiting both animal welfare and production timelines (45). Dot blot and neutralization assays confirmed the specificity of the antibodies, with measurable activity at high dilutions (up to 1:20,480) (45). These results underscore the potential of IgY as a scalable and effective agent for RSV-related prophylaxis, possibly extending to human applications in the future.

### 2.4 Structural, functional, and practical limitations of IgY

Despite their growing promise as next-generation immunotherapeutics, IgY exhibits intrinsic limitations that affect their pharmacological performance, manufacturability, and translational potential. Structurally, IgY lacks the flexible hinge region found in mammalian IgG, which can limit antigen-binding adaptability (5, 46). IgY is also highly sensitive to pH extremes and repeated freeze–thaw cycles, resulting in loss of activity under acidic or unstable storage conditions (47). Functionally, reviews report pH-dependent neutralization efficiency and occasionally limited *in-vivo* stability (5, 47). From a practical perspective, large-scale production faces hurdles such as purification complexity, batch variability, and high cost for monoclonal formulations (46) (Constantin et al., 2020). While polyclonal IgY is relatively inexpensive, scaling to clinical-grade monoclonal IgY remains challenging due to standardization and regulatory constraints (48).

In summary, avian IgY antibodies have shown considerable promise in the prophylaxis and treatment of several major respiratory viral infections. Immunization with either inactivated whole virus (e.g., H1N1, BRSV, Influenza B), or recombinant antigens (e.g., SARS-CoV-2 RBD), results in high-yield antibody production. The use of IgY offers a rapid, low-cost, and scalable countermeasure—particularly suitable for outbreak response, mucosal delivery, and passive protection, especially in vulnerable populations where vaccines are delayed or less effective. Prospectively, the integration of IgY-based prophylaxis into pandemic-preparedness frameworks could complement existing vaccine programs, especially for frontline workers or immunocompromised individuals. Advances in recombinant-antigen design, large-scale purification, and freeze-drying technologies are now enabling shelf-stable nasal or oral IgY therapeutics that can be stockpiled and deployed rapidly during viral outbreaks. As regulatory pathways for biologics evolve, IgY antibodies may soon occupy a niche similar to monoclonal antibodies, offering safe, ethical, and scalable immunotherapy derived from a renewable source.

## 3 Camelid nanobodies and shark VNARs (single-domain antibodies)

Single-domain antibodies (sdAbs) represent an innovative class of antibody fragments composed solely of heavy-chain variable domains, capable of binding antigens with high specificity and affinity (49). Among them, two prominent representatives have emerged: camelid- nanobodies (derived from variable domain of the heavy chain; (VHH)) and shark-derived VNARs (variable new antigen receptors derived from the IgNAR). Despite their similar monomeric formats and molecular weights (~12–15 kDa), these molecules evolved independently and exhibit distinct structural features that define their antigen-binding behavior.

Camelid nanobodies originate from heavy-chain-only antibodies found in camels, llamas, and alpacas. These VHH domains are soluble, thermally stable, and amenable to microbial expression, making them highly suitable for recombinant production (50). A defining feature of nanobodies is their elongated complementarity-determining region 3 (CDR3), which enables them to reach deeply recessed or hidden epitopes, regions often inaccessible to conventional IgG antibodies (51).

In parallel, VNARs are derived from the IgNAR isotype found in cartilaginous fish, including sharks (52). Their unique stabilization mechanism relies on inter-CDR disulfide bonds, particularly between CDR1 and CDR3 (53). This configuration imparts exceptional stability under acidic or denaturing conditions, allowing VNARs to maintain binding activity in low-pH environments or in the presence of hydrophobic interfaces (53).

Both nanobodies and VNARs exhibit remarkable adaptability in recognizing a broad spectrum of antigens—from small molecules and enzymes to viral glycoproteins. Their compact size not only allows for deep antigen penetration but also supports multivalent and multispecific engineering, where several paratopes can be fused to enhance binding strength, broaden target coverage, or modulate pharmacokinetics.

### 3.1 Unique properties of camelid nanobodies and shark VNARs: comparative advantages to mammalian IgG

Unlike conventional antibodies like IgG, which are large and complex with two heavy and two light chains, nanobodies and VNARs are single-domain antibodies (sdAbs) that simplify this structure significantly. Nanobodies are derived from camelids (such as camels and llamas) and consist of only VHH, while VNARs come from sharks and originate from a unique type of immunoglobulin known as IgNAR (54, 55). Both are extremely small, yet retain high specificity and affinity for their targets.

The compact design of nanobodies and VNARs enables them to access recessed or hidden antigenic sites that are often inaccessible to traditional antibodies (6, 8, 51). Their small size also allows better tissue penetration and faster clearance from the body, which can be advantageous in certain therapeutic contexts. Nanobodies, in particular, feature an extended CDR3 loop that enables them to engage with hard-to-reach or deeply buried epitopes, such as enzyme pockets or viral surface grooves (51). Indeed, nanobodies excel at binding to concave and recessed surfaces on antigens, where larger antibodies typically fail due to steric hindrance. Their flexible, elongated CDR3 loops can dive into grooves and clefts (51). Nanobodies are also effective at recognizing cryptic or hidden epitopes. Studies show that VHH nanobodies can bind enzyme active sites, such as the catalytic cleft of lysozyme, through their long CDR3 loops (56). In infectious disease research, nanobodies targeting *Clostridium difficile* toxin components blocked enzymatic activity by penetrating the NAD-binding site (57). In the context of SARS-CoV-2, bispecific nanobodies bn03 which showed broad neutralizing activity by targeting conserved, hidden regions within the spike protein, including sites shielded in trimeric structures of RBD (58). Overall, the unique structure and biochemical properties of nanobodies and VNARs offer compelling advantages for immunotherapy, diagnostics, and biotechnology especially when targeting viral antigens or delivering antibodies to difficult-to-reach tissues.

### 3.2 Practical advantages of camelid nanobodies and shark VNARs production

In terms of production efficiency, these single-domain antibodies can be efficiently expressed in microbial systems like *E. coli* and yeast, which makes them attractive for large-scale, cost-effective manufacturing (6). Their frameworks are rich in hydrophilic residues, especially in framework region 2, enhancing their solubility and resistance to aggregation (59). In addition, nanobodies and VNARs are naturally more stable than conventional antibodies, retaining functionality across a wide range of pH, temperature, and solvent conditions (11, 50). This makes them ideal candidates for field-deployable diagnostics, oral or nasal delivery formulations, and applications in resource-limited settings (11). However, their small size also means they are rapidly cleared from circulation. To address this, they can be engineered with Fc domains, PEGylated, or fused with albumin to extend their half-life (8, 60–62). These capabilities make nanobodies and VNARs exceptionally versatile tools for the development of immunotherapies against viral infections.

### 3.3 Therapeutic applications of camelid nanobodies against respiratory viruses

Influenza viruses undergo frequent antigenic drift, especially in their hemagglutinin (HA) proteins, posing challenges for conventional vaccine and antibody approaches. Nanobodies, due to their structural plasticity and small size, provide promising alternatives capable of targeting conserved and often sterically shielded regions on the HA protein (63). A study designed multidomain antibodies (MDAbs) by fusing four camelid-derived nanobodies targeting distinct conserved regions across both influenza A and B strains (63). The constructs MD3606 and MD2407 target HA stem regions and receptor-binding sites of several influenza A and B strains. These MDAbs were delivered intranasally in mice using adeno-associated virus (AAV) vectors, achieving robust protection against lethal challenges with H1N1 and H7N9 (63). The combined targeting of stem and receptor-binding domains blocks both viral attachment and membrane fusion, critical steps in viral entry (63). Another study demonstrated the therapeutic potential of nanobody E10 which can bind a lateral patch on the HA head domain, a cryptic site shielded from immune recognition in typical immune responses (64). This site, centered around residues K166 and S167, is conserved among H1, H3, and H7 subtypes, providing a route for broad subtype coverage (64). A study using an Fc-fused nanobody targeting HA2 reported full protection in animal models exposed to lethal IAV doses (65). Collectively, these studies highlight the unique advantage of nanobodies in penetrating conserved yet sterically hindered antigenic pockets on HA that are inaccessible to conventional monoclonal antibodies. Because of their small (≈15 kDa) monomeric size and extended CDR3 loop, VHHs can recognize epitopes buried at the interface of HA monomers or within the fusion stem, regions that remain conserved across multiple influenza subtypes. In this way, they hold strong potential as universal influenza countermeasures when combined into multidomain or multiepitope constructs.

Nanobodies also show promise against less conventional targets like neuraminidase (NA) and matrix protein M2 (66, 67). Although traditionally seen as non-neutralizing, NA-binding nanobodies have been shown to delay viral spread by preventing virion release. One study showed that NA-specific nanobodies protected mice from lethal H5N1 challenge, including oseltamivir-resistant strains (66). Similarly, nanobodies against the M2 ion channel reduced viral replication *in vivo*, though without full protection (67). These emerging strategies broaden the antiviral scope of nanobodies and demonstrate their flexibility in targeting dynamic and elusive viral epitopes. Moreover, the inherent stability of camelid VHHs, maintaining structure after lyophilization, high temperature exposure, and aerosolization, makes them ideal for formulations such as intranasal sprays or inhalable powders. Using AAV-encoded nanobodies have demonstrated long-term expression in respiratory epithelia, enabling months-long mucosal protection with a single administration (63). Such delivery innovations could transform prophylaxis during influenza epidemics, particularly among immunocompromised or elderly populations.

The SARS-CoV-2 S protein is a trimeric glycoprotein that plays a central role in viral entry into host cells, making it a prime target for therapeutic intervention. Nanobodies have been developed against various regions of this protein to block infection through multiple mechanisms (6). Key regions of interest include the receptor-binding domain (RBD) and the N-terminal domain (NTD). Several nanobodies, such as VHH72, mNb6, and Ty1, have demonstrated high-affinity binding to the RBD, effectively blocking the virus’s ability to interact with the ACE2 receptor (68–70). Other nanobodies like XG2v046 and XGv280 target the NTD. By destabilizing the prefusion spike trimer, they interfere with viral entry mechanisms (71). Another study introduced multivalent nanobody constructs that fuse multiple RBD-specific nanobodies into a single molecule (72). These include homotrimeric forms (e.g., EEE) that enhance avidity and potency, and biparatopic forms (e.g., VE and EV) that bind two non-overlapping epitopes on the RBD (72). The biparatopic design has proven especially effective at neutralizing diverse SARS-CoV-2 variants by reducing escape through simultaneous dual-epitope engagement (72). Further engineering has yielded aerosolized and thermostable nanobodies that remain active at room temperature and under shear stress during nebulization (73, 74). This physicochemical robustness allows their use in portable, non-invasive delivery systems suitable for both prophylaxis and post-exposure therapy. In animal models, intranasally administered nanobody cocktails provided rapid viral clearance in the upper airways, an advantage not typically achieved by parenterally administered monoclonals (75, 76).

A notable example is the bispecific nanobody bn03 (58). Cryo-EM analyses revealed that bn03 binding locks the RBD in a neutralized conformation, achieving broad activity against Omicron subvariants (BA.1, BA.2, BA.4/5) and other variants of concern (58). Furthermore, its small size and high stability allowed formulation into inhalable aerosols, which showed therapeutic efficacy in murine models by reducing viral loads in both upper and lower respiratory tracts (58). This work underscores the unique adaptability of nanobodies in rapidly evolving viral outbreaks.

Beyond RBD neutralization, recent studies have identified nanobodies targeting conserved epitopes within the S2 fusion core, capable of cross-reacting with SARS-CoV-1 and MERS-CoV (62, 77). Such broadly reactive nanobodies could provide the foundation for pan-sarbecovirus therapeutics. Their modularity allows combination into multiepitope constructs that retain potency despite emerging mutations, a property already being explored in clinical-stage inhalable VHH therapies (76, 78).

Camelid single-domain antibodies have been extensively evaluated for RSV. Nanobodies directed against the prefusion conformation of the RSV fusion (F) glycoprotein exhibit potent neutralizing activity and therapeutic efficacy in both prophylactic and post-infection models. Intranasal and inhaled VHH constructs (bivalent, trimeric, and Fc-fused formats) have shown marked reductions in lung viral titers, inflammation, and disease severity, confirming their suitability as inhalable antiviral biologics for respiratory infections (78–81). Beyond RSV, recent investigations have extended single-domain antibody applications to human metapneumovirus (HMPV), another pneumovirus closely related to RSV. Neutralizing nanobodies have been developed against conserved trimer-interface epitopes on the HMPV F protein, providing protection *in vivo* (82). Of note, a study has revealed cross-neutralizing antibodies targeting structurally conserved prefusion-F epitopes shared between RSV and HMPV (83). These findings suggest that conserved antigenic sites across pneumoviruses can be exploited to design broad-spectrum single-domain antibody therapeutics, and that analogous epitope regions could serve as targets for IgY- or VNAR-based platforms in future mucosal immunotherapy development.

Collectively, the exceptional camelid nanobodies solubility, deep-epitope recognition, and facile engineering for bispecific or multivalent constructs confer unique advantages over conventional antibodies (**Table 2**). When coupled with intranasal or aerosol delivery routes, nanobodies represent a next-generation class of biologics capable of preventing infection at its point of entry, offering rapid, variant-resilient protection against viral respiratory infections (63, 73, 75).

**Table 2:** Summary of camelid single-domain antibodies (nanobodies/VHHs) targeting epitopes of respiratory viruses, demonstrating neutralization and intranasal delivery potential.

Study/year	Viral target	VHH target antigen	Treatment timing	Outcome/notes
Ibañez et al., 2011 (84)	Influenza A (H5N1)	HA (hemagglutinin)	Therapeutic (mice)	Anti-HA VHHs neutralized H5N1 *in vitro* and protected mice *in vivo*.
Barbieri et al., 2024 (85)	Influenza A (H1 subtype)	HA (monomeric VHH)	Prophylactic (mice)	Single VHH conferred prophylactic immunity against H1 viruses.
Hwang et al., 2024 (86)	Influenza A (H1N1)	HA	Therapeutic (mice)	Significant therapeutic effect at 0.5 mg/kg post-infection.
Schepens et al., 2011 (79)	RSV	F (fusion glycoprotein, prefusion-specific)	Prophylactic (intranasal, mice)	Intranasal bivalent VHH protected mice and reduced lung inflammation.
Detalle et al., 2016 (80)	RSV	F (trimeric VHH; ALX-0171)	Therapeutic (inhaled; preclinical)	Potent neutralization vs RSV A/B; basis for inhaled infant therapy.
Mora et al., 2018 (81)	RSV	F (ALX-0171)	Therapeutic (nebulized; newborn lambs)	Greatly reduced lung viral load & pathology in lamb RSV model.
Cunningham et al., 2021 (78)	RSV	F (ALX-0171)	Therapeutic (inhaled; infants, Phase II)	Safe but no clinical improvement vs placebo despite antiviral signals.
Ballegeer et al., 2023 (82)	HMPV	Fusion (F) glycoprotein (trimer-interface epitope)	Therapeutic (*in vitro* and *in vivo*)	Provided potent viral neutralization and protection in animal models.
Zhao et al., 2018 (87)	MERS-CoV	Spike RBD (novel VHHs)	Prophylactic/Therapeutic (*in vitro*; animal data described)	Potent anti-MERS VHHs with cross-strain activity; therapeutic potential.
Raj et al., 2018 (77)	MERS-CoV	Spike RBD (VHHs; chimeric HCAbs)	Therapeutic (hDPP4-Tg mice)	Protected mice from lethal MERS-CoV; reduced lung titers/pathology.
Wrapp et al., 2020 (88)	MERS-CoV & SARS-CoV-1/2 (pseudovirus)	Spike RBD (e.g., VHH-72)	Prophylactic/Therapeutic (*in vitro*; Fc-fusion enhances)	Structural/functional cross-neutralization; foundation for therapeutics.
Huo et al., 2021 (74)	SARS-CoV-2	Spike RBD (homotrimeric VHHs)	Prophylactic/Therapeutic (hamsters)	Intranasal VHH trimers reduced weight loss, lung viral burden & pathology.
Wu et al., 2021 (89)	SARS-CoV-2	Spike RBD (bispecific Nb15-NbH-Nb15)	Prophylactic & Therapeutic (intranasal; hACE2 mice)	Effective protection in both settings via intranasal dosing.
Esparza et al., 2022 (73)	SARS-CoV-2	Spike RBD (NIH-CoVnb-112)	Therapeutic (nebulized; hamsters)	Reduced weight loss, viral burden, and lung pathology after nebulization.
Wu et al., 2022 (75)	SARS-CoV-2 (Delta)	Spike RBD (Nb22)	Prophylactic & Therapeutic (intranasal; hACE2 mice)	Protection even when dosed 7 days pre-challenge; strong post-exposure efficacy.
Aksu et al., 2024 (76)	SARS-CoV-2 (multiple variants)	Spike RBD (multivalent VHHs; aerosol)	Prophylactic & Therapeutic (hamsters; aerosolized)	Before or 24 h after infection, aerosols reduced viral load, weight loss, and pathogenicity.
Liu et al., 2024 (90)	SARS-CoV-2 (WT & variants)	Spike RBD (VHH60)	Prophylactic/Therapeutic (cells & mice)	Protected mice and blocked entry of multiple variants; broad activity.

### 3.4 Therapeutic Applications of Shark VNARs Against Respiratory Viruses

The unique structural features and binding versatility of vNARs position them as a promising and scalable platform for the development of novel immunotherapeutics targeting conserved membrane proteins in respiratory viruses (**Table 3**).

**Table 3:** Summary of shark-derived variable new antigen receptors (VNARs) with neutralizing activity against respiratory viruses.

Study/year	Viral target	VNAR/IgNAR target Antigen/Epitope	Experiments	Outcome/notes
Yu et al., 2023 (91)	Influenza A (M2 ion channel)	Tetrameric M2 protein	*In vitro* mechanistic study, binding to membrane-embedded M2; allosteric inhibition of proton channel activity in wild-type and S31N amantadine-resistant strains	AM2H10 exhibited specific binding to native M2 tetramer (not linear M2e peptides), disrupted ion conductance via allosteric conformational changes, and retained activity against resistant mutants; affinity maturation and multimerization enhanced avidity and stability.
Ubah et al., 2021 (92)	SARS-CoV-2	RBD (distinct epitopes)	*In vitro* neutralization & structural analysis	Two VNARs (3B4, 2C02) neutralize live SARS-CoV-2 and pseudovirus; crystallography reveals distinct mechanisms.
Feng et al., 2022 (93)	SARS-related coronaviruses (broad)	RBD (conserved region)	*In vitro* binding & neutralization	Two shark VNARs broadly bind and neutralize sarbecovirus RBDs.
Chen et al., 2023 (94)	SARS-CoV-2/broader sarbecoviruses	RBD (two non-overlapping conserved epitopes)	*In vivo* challenge (K18-hACE2 mice)	Shark “ShAb” IgNAR-Fc chimeras cross-neutralize SARS-CoV-2 variants and protect mice.
Liu et al., 2024 (95)	SARS-CoV-2 (WT + variants)	RBD (Spike) — shark VNAR R1C2	Binding assays, neutralization (*in vitro*)	R1C2 binds Spike RBD of wild type and Omicron variants.
Feng et al., 2025 (96)	SARS-CoV-2 (Omicron subvariants + sarbecoviruses)	Spike **S2/HR1 region** — highly conserved epitope	Intranasal prophylaxis (*in vivo*)	VNAR 79C11 neutralizes Omicron subvariants, SARS-CoV-1, pangolin CoV. Intranasal instillation prevents infection of XBB in mice.

Researchers have explored shark-derived vNARs as next-generation antiviral agents against influenza. The vNAR AM2H10 was isolated from Chiloscyllium plagiosum (bamboo shark) following immunization with nanodisc-reconstituted tetrameric M2 protein (91). This approach preserved the native membrane-bound structure of M2, enhancing antigenicity and enabling the identification of functional binders (91). AM2H10 exhibited high specificity for the native M2 tetramer, rather than linear M2e peptides, and demonstrated potent inhibition of proton channel activity in both wild-type and amantadine-resistant (S31N) M2 channels (91). This study represented the first reported vNAR with antiviral activity against influenza A, offering both broad-spectrum potential and resistance-overcoming capability (91). This discovery was particularly significant because it illustrated how VNARs, unlike conventional monoclonal antibodies, can bind to recessed or transmembrane epitopes, regions of viral ion channels and fusion proteins that are typically inaccessible. The exceptional paratope plasticity of VNARs, defined by their elongated CDR3 loops and truncated β-sandwich scaffold, allows these antibodies to probe concave epitopes, allosteric sites, and channel pores that are beyond the reach of traditional IgG or even camelid nanobodies (6, 59). This structural property gives VNARs an edge in tackling highly conserved but cryptic viral targets, such as M2, HA stem, and fusion subunits.

In the context of the COVID-19 pandemic, where viral evolution has continually outpaced many conventional antibody therapies, shark-derived VNARs have emerged as promising next-generation therapeutics (7). Studies have demonstrated the potent neutralizing capacity of VNARs across a wide range of SARS-CoV-2 variants, including Alpha, Beta, Delta, and Omicron (7). VNARs have been isolated from both immune and synthetic libraries using phage display as the dominant selection platform, with several reengineered as Fc-fusion constructs to enhance avidity, half-life, and therapeutic potency. Notably, clones such as 20G6-Fc and SP240 have shown broad-spectrum neutralization in both *in vitro* and *in vivo* models (93). Some VNARs, such as 3B4, 2C02, and 4C10 have demonstrated strong binding and neutralizing activity (IC_50_< 10 nM) against both SARS-CoV-2 and SARS-CoV-1 *in vitro*. Furthermore, these VNARs also neutralized WIV1-CoV, a pre-emergent zoonotic virus, with 3B4 additionally capable of neutralizing MERS-CoV pseudovirus, likely due to its small size and protruding CDR3, enabling access to conserved β-coronavirus epitopes. Structural analyses revealed that 3B4 and 2C02 bind distinct, non-overlapping sites on the RBD, away from the ACE2 interface, and hence the study proposes a therapeutic cocktail comprising 3B4 and 2C02 based on their complementary binding profiles and cross-variant neutralization potential (92). Subsequent cryo-EM analyses of newer VNARs such as 79C11 and R1C2 revealed binding to conserved epitopes within the spike S2 or HR1 regions, conferring cross-neutralization against SARS-CoV-1, Omicron subvariants, and even sarbecoviruses found in bats and pangolins (95, 96). In murine models, intranasal administration of VNAR-Fc constructs led to significant reductions in lung viral loads and prevented alveolar inflammation, underscoring their potential for mucosal immunotherapy (94, 96).

Because VNARs are only 12–15 kDa, among the smallest naturally occurring antibody fragments, they display excellent thermostability, resistance to pH extremes, and compatibility with aerosolization and lyophilization. This makes them highly suitable for pulmonary delivery, as demonstrated by recent intranasal or aerosolized VNAR formulations that retained full neutralizing activity after nebulization (96). Moreover, their simple genetic structure enables rapid selection and optimization from synthetic libraries within weeks, drastically shortening discovery timelines during emerging outbreaks.

Altogether, these findings underscore the clinical potential of VNARs as highly adaptable, modular antibody formats for combating respiratory viruses. Their unique structure, compatibility with diverse engineering strategies, and rapid discovery timelines from synthetic libraries position them as a powerful complement to current antiviral platforms, especially in the face of mutational drift and vaccine resistance. Moving forward, VNARs could serve as ideal building blocks for hybrid biologics, such as bispecific VNAR–VHH or VNAR–IgY fusion constructs, combining the deep-epitope access of shark antibodies with the neutralization breadth and mucosal stability of avian and camelid systems (90, 94, 95). Their potential for low-cost microbial expression and high yield also supports future large-scale manufacturing, paving the way toward accessible, thermostable, and variant-resilient therapeutics for global respiratory health emergencies (92, 96).

### 3.5 Structural, functional, and practical limitations of camelid nanobodies and shark VNARs

Camelid nanobodies and shark VNARs share a compact, single-domain structure that grants unique advantages but introduces common disadvantages. Structurally, their small molecular mass (~12–15 kDa) leads to rapid renal clearance and short serum half-life, often necessitating Fc fusion or PEGylation to prolong circulation (8, 97). However, these modifications increase molecular size, limit mucosal delivery (e.g., intranasal), and elevate production cost. Both nanobodies and VNARs are prone to aggregation during bacterial or yeast expression, and shark VNARs in particular exhibit complex disulfide-bond patterns that can hinder correct folding (98).

Functionally, single-domain antibodies show reduced cross-reactivity across viral variants, particularly in highly variable spike regions (S1 domain), and cannot recruit Fc-mediated immune functions unless engineered (97). Immunogenicity remains a potential risk when derived from non-human species, especially sharks.

From a practical standpoint, the main challenges include instability during storage, loss of bacterial production efficiency after Fc fusion, high PEGylation cost, and limited industrial infrastructure for VNAR manufacturing (8). Regulatory pathways for marine- or camelid-derived therapeutic antibodies are still under development, representing a major translational bottleneck. In summary, while single-domain antibodies (VHHs and VNARs) provide exciting opportunities for respiratory antiviral therapy, their successful clinical deployment will depend on overcoming half-life limitations, stability optimization, and manufacturing scalability through continued bioengineering and regulatory innovation.

## 4 Conclusions and future directions

The evolution of antibody formats beyond the classical IgG paradigm has opened new horizons in the fight against viral infections. This review has explored the structural and functional diversity of alternative antibody systems derived from birds, camelids, and sharks, underscoring their capacity to overcome some of the intrinsic limitations of conventional antibodies. IgY, nanobodies, and VNARs represent distinct immunological innovations shaped by divergent evolutionary pressures, yet they converge on a common theme: adaptability to hostile environments, unique epitope recognition, and amenability to novel delivery platforms. Their demonstrated efficacy in neutralizing respiratory viruses, including influenza strains, coronaviruses, and paramyxoviruses, highlights their translational promise for both prophylactic and therapeutic applications. These antibody types also hold immense potential in the development of mucosal immunotherapies, a domain where traditional IgG-based biologics often falter due to poor stability and immunogenic complications.

Despite these advances, the clinical and commercial realization of these antibody classes remains at a formative stage. The immunogenicity of non-human-derived sequences, challenges in regulatory standardization, and the absence of large-scale manufacturing pipelines tailored to these molecules are non-trivial hurdles. Additionally, while their small size and unique architectures offer delivery advantages, they also pose pharmacokinetic limitations, including rapid systemic clearance. Overcoming these barriers will require further bioengineering innovations, such as half-life extension technologies, improved formulation strategies, and scalable recombinant production systems. It is equally essential to generate robust clinical data to validate their safety and efficacy across diverse populations and viral contexts. Nonetheless, as viral pathogens continue to evolve and evade traditional immune countermeasures, the strategic inclusion of IgY, nanobodies, and VNARs in the antiviral toolkit offers a resilient and versatile approach to next-generation immunotherapy. Their integration into mainstream biomedical pipelines will not only diversify therapeutic modalities but also enhance our preparedness for future infectious disease threats.
